# Ethyl 1-acetyl-1*H*-indole-3-carboxyl­ate

**DOI:** 10.1107/S1600536809025379

**Published:** 2009-07-08

**Authors:** Tasneem Siddiquee, Shahid Islam, Dennis Bennett, Matthias Zeller, Mahmun Hossain

**Affiliations:** a Department of Chemistry, Boswell Science Complex, Tennessee State University, Nashville, 3500 John A Merritt Blvd, Nashville, TN 37209, USA; b Department of Chemistry and Biochemistry, University of Wisconsin-Milwaukee, 3210 N Cramer Street, Milwaukee, WI 53211, USA; c Youngstown State University, Department of Chemistry, One University Plaza, Youngstown Ohio 44555-3663, USA

## Abstract

The title compound, C_13_H_13_NO_3_, was synthesized by acetyl­ation of ethyl 1*H*-indole-3-carboxyl­ate. The aromatic ring system of the mol­ecule is essentially planar, but the saturated ethyl group is also located within this plane and the overall r.m.s. deviation from planarity is only 0.034 Å. Pairs of C—H⋯O inter­actions connect mol­ecules into chains along the diagonal of the unit cell. Mol­ecules also form weakly connected dimers *via* π⋯π stacking inter­actions of the indole rings with centroid–centroid separations of 3.571 (1) Å. C—H⋯π inter­actions between methyl­ene and methyl groups and the indole and benzene ring complete the directional inter­molecular inter­actions found in the crystal structure.

## Related literature

For the biological properties of tryptophan derivatives, see: Ma *et al.* (2001 ▶); Zhou *et al.* (2006 ▶); Zhao, Smith *et al.* (2002 ▶); Zhao, Liao & Cook (2002 ▶). For synthetic procedures towards tryptophan-like compounds, see: Ager & Laneman (2004 ▶); Amir-Heidari *et al.* (2007 ▶); Carlier *et al.* (2002 ▶); Hengartner *et al.* (1979 ▶); Moriya *et al.* (1980 ▶). For the synthesis of 2-acetamido-3-eth­oxy-3-oxopropanoic acid, see: Hellmann *et al.* (1958 ▶). For NMR data for the title compound, see: Reimann *et al.* (1990 ▶).
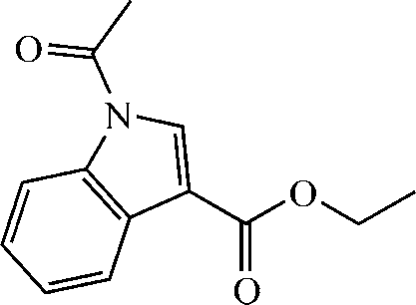


## Experimental

### 

#### Crystal data

C_13_H_13_NO_3_
*M* *_r_* = 231.24
Triclinic, 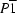

*a* = 7.519 (1) Å
*b* = 8.479 (1) Å
*c* = 10.187 (2) Å
α = 97.38 (1)°
β = 95.78 (2)°
γ = 114.28 (1)°
*V* = 578.58 (15) Å^3^
*Z* = 2
Mo *K*α radiation
μ = 0.10 mm^−1^
*T* = 296 K
0.51 × 0.41 × 0.20 mm

#### Data collection

Siemens P4 diffractometer
Absorption correction: multi-scan [*XSCANS* (Siemens, 1996 ▶) and *XPREP* (Siemens, 1994 ▶)] *T*_min_ = 0.823, *T*_max_ = 0.981
2536 measured reflections
2027 independent reflections
1696 reflections with *I* > 2σ(*I*)
*R*_int_ = 0.019
3 standard reflections every 97 reflections intensity decay: <1%

#### Refinement

*R*[*F* ^2^ > 2σ(*F* ^2^)] = 0.042
*wR*(*F* ^2^) = 0.120
*S* = 1.09
2027 reflections
155 parameters
H-atom parameters constrained
Δρ_max_ = 0.17 e Å^−3^
Δρ_min_ = −0.18 e Å^−3^

### 

Data collection: *XSCANS* (Siemens, 1996 ▶); cell refinement: *XSCANS*; data reduction: *XSCANS*; program(s) used to solve structure: *XPREP* (Siemens 1994 ▶) and *SHELXTL* (Sheldrick, 2008 ▶); program(s) used to refine structure: *SHELXTL*; molecular graphics: *SHELXTL*; software used to prepare material for publication: *SHELXTL*.

## Supplementary Material

Crystal structure: contains datablocks I, global. DOI: 10.1107/S1600536809025379/bh2233sup1.cif

Structure factors: contains datablocks I. DOI: 10.1107/S1600536809025379/bh2233Isup2.hkl

Additional supplementary materials:  crystallographic information; 3D view; checkCIF report

## Figures and Tables

**Table 1:** Hydrogen-bond geometry (Å, °)

*D*—H⋯*A*	*D*—H	H⋯*A*	*D*⋯*A*	*D*—H⋯*A*
C2—H2⋯O3^i^	0.93	2.61	3.296 (2)	131
C5—H5⋯O1^ii^	0.93	2.64	3.273 (2)	125
C12—H12*B*⋯*Cg*1^iii^	0.96	2.95	3.618 (3)	127
C13—H13*B*⋯*Cg*2^iii^	0.96	2.78	3.587 (3)	142

Symmetry codes: (i) 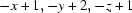; (ii) 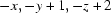; (iii) 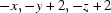. *Cg*1 is the centroid of the N1,C1,C6–C8 pyrrole ring and *Cg*2 is the centroid of the C1–C6 phenyl ring.

**Fig. 1.**
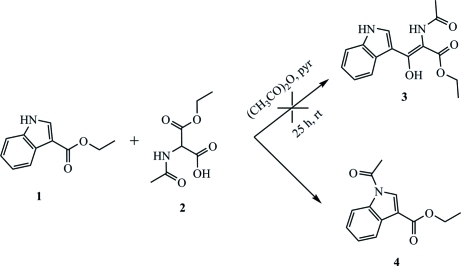
Synthesis of the title compound (4).

**Fig. 2.**
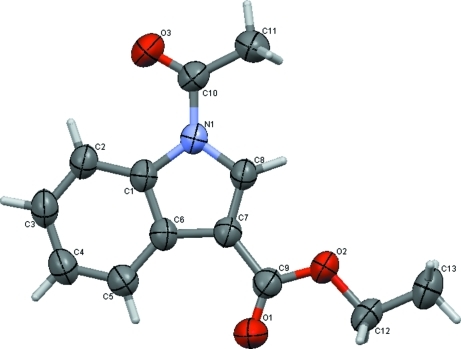
Thermal ellipsoid plot of the title compound with the atom labeling scheme. Displacement ellipsoids are shown at the 50% probability level and H atoms are shown as capped sticks.

**Fig. 3.**
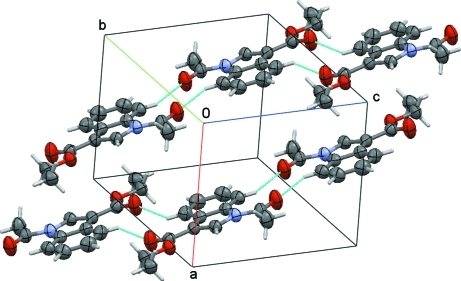
Packing view of the title compound showing C—H···O interactions (blue lines).

## supplementary crystallographic information

### Comment

Indole substituted at 3-position leads to variety of compounds that are precursors to biologically active important alkaloids. One of the most important compounds of this type is tryptophan, which possesses anticancerous, antimalarial, antiamoebic, and antihypertensive activities (Ma *et al.*, 2001; Zhou *et al.*, 2006; Zhao, Smith *et al.* 2002; Zhao, Liao, & Cook, 2002). α,β-Dehydroaminoacid esters (*e.g.*1, Fig. 1) are precursors to synthesizing tryptophan derivatives, which upon hydrogenation yield optically active tryptophan and its analogues (Ager & Laneman, 2004).

α,β-Dehydroamino acid esters were also synthesized using Erlenmeyer condensation (Amir-Heidari *et al.*, 2007), Schmidt olefinations (Carlier *et al.*, 2002), condensation of indole aldehyde with acetylamino malonic acid ester (Hengartner *et al.*, 1979), and Knoevenagel-type condensation (Moriya *et al.* 1980). One such effort to synthesize hydroxyl dehydrotryptophan (3) from indoleester (1) using mono acid malonic ester (2) and aceticanhydride-pyridine mixture (Fig. 1) proved to be unsuccessful. The reaction resulted in 1*H*-indole-3-carboxylic acid-*N*-acetylethyl ester (4) instead. We rationalize that it is the electron withdrawing effect of the ester group which increases the acidity of the molecule. Consequently, in presence of a base, like pyridine, deprotonation and introduction of an acylium ion may occur. In this article we report the crystal structure of this compound.

The structure of the title compound is shown in Figure 2. The aromatic ring system of the molecule is essentially planar, but also the saturated ethyl group is located within this plane and the overall r.m.s. deviation from planarity is only 0.034 Å. Pairs of C—H···O interactions connect molecules into chains along the diagonal of the unit cell (Fig. 3). Molecules form weakly connected dimers *via*π···π stacking interactions of the indole rings with centroid to centroid distances of 3.571 (1) Å [symmetry operator for the second indole ring: (iii) 1 - *x*, 2 - *y*, 2 - *z*]. C—H···π interactions between methylene and methyl groups and the indole and benzene ring complete the range of intermolecular interactions [C12—H12B···*Cg*1^iii^ = 2.95 Å, *X*—H···*Cg*1^iii^ = 127°, *X*···*Cg*1^iii^ = 3.618 (3) Å; C13—H13B···*Cg*2^iii^ = 2.78 Å, *X*—H···*Cg*2^iii^ = 142°, *X*···*Cg*2^iii^ = 3.587 (3) Å; *Cg*1 and *Cg*2 are the centroids of the indole and the benzene rings, respectively].

### Experimental

2-Acetamido-3-ethoxy-3-oxopropanoic acid (one of the starting materials) was prepared from acetylamino malonic acid diethylester following the process developed by Hellmann *et al.* (1958). The title compound was prepared as follows: to a mixture of 0.37 g (1.97 mmol) of the indole ester ethyl 1*H*-indole-3-carboxylate, 1.1 g (5.9 mmol) of 2-acetamido-3-ethoxy-3-oxopropanoic acid, and 4.54 ml of pyridine was added at 288 K (15 °C) over 15 minutes 1.6 ml of acetic anhydride. The reaction mixture turned yellow and was stirred at 333 K (60 °C) for 3 h. An additional 0.18 g (0.9 mmol) of ethyl acetamido malonate was added and stirring was continued for 22 h. Ice (10 ml) was added, and the mixture was stirred for 2 h and then diluted with 20 ml of water. The resulting solution was extracted with EtOAc (2 × 20 ml), the combined organic layer was dried with anhydrous Na_2_SO_4_ and the solvent was removed under reduced pressure. 0.4 g (99%) of 1*H*-indole-3-carboxylicacid-*N*-acetyl ethyl ester was isolated. ^1^HNMR CDCl_3_δ (p.p.m.): 8.70–8.50 (m,1H and 2H), 7.60–7.30 (m, 2H), 4.45 (q, *J* = 7 Hz, OCH_2_CH_3_), 2.70 (s, COCH_3_), 1.45 (t, *J* = 7 Hz, OCH_2_CH_3_). The NMR data agree with those reported previously (Reimann *et al., *1990). Crystals suitable for X-ray structural analysis were obtained by recrystallization form ethanol in a refrigerator.

### Refinement

All hydrogen atoms were added in calculated positions with a C—H bond distances of 0.97 (methylene), 0.93 (aromatic) and 0.96 Å (methyl). They were refined with isotropic displacement parameters *U*_iso_ of 1.5 (methyl) or 1.2 times *U*_eq_ (all others) of the adjacent carbon atom.

### Crystal data

**Table:**

C_13_H_13_NO_3_	*Z* = 2
*M**_r_* = 231.24	*F*(000) = 244
Triclinic, *P*1	*D*_x_ = 1.327 Mg m^−^^3^
Hall symbol: -P 1	Mo *K*α radiation, λ = 0.71073 Å
*a* = 7.519 (1) Å	Cell parameters from 23 reflections
*b* = 8.479 (1) Å	θ = 3.7–11.4°
*c* = 10.187 (2) Å	µ = 0.10 mm^−^^1^
α = 97.38 (1)°	*T* = 296 K
β = 95.78 (2)°	Block, colourless
γ = 114.28 (1)°	0.51 × 0.41 × 0.20 mm
*V* = 578.58 (15) Å^3^	

### Data collection

**Table:**

Siemens P4 diffractometer	1696 reflections with *I* > 2σ(*I*)
Radiation source: fine-focus sealed tube	*R*_int_ = 0.019
graphite	θ_max_ = 25.0°, θ_min_ = 2.1°
2θ/ω scans	*h* = −8→1
Absorption correction: multi-scan [*XSCANS* (Siemens 1996) and *XPREP* (Siemens, 1994)]	*k* = −9→9
*T*_min_ = 0.823, *T*_max_ = 0.981	*l* = −12→12
2536 measured reflections	3 standard reflections every 97 reflections
2027 independent reflections	intensity decay: <1%

### Refinement

**Table:**

Refinement on *F*^2^	Secondary atom site location: difference Fourier map
Least-squares matrix: full	Hydrogen site location: inferred from neighbouring sites
*R*[*F*^2^ > 2σ(*F*^2^)] = 0.042	H-atom parameters constrained
*wR*(*F*^2^) = 0.120	*w* = 1/[σ^2^(*F*_o_^2^) + (0.0647*P*)^2^ + 0.0738*P*] where *P* = (*F*_o_^2^ + 2*F*_c_^2^)/3
*S* = 1.09	(Δ/σ)_max_ = 0.001
2027 reflections	Δρ_max_ = 0.17 e Å^−^^3^
155 parameters	Δρ_min_ = −0.18 e Å^−^^3^
0 restraints	Extinction correction: *SHELXTL* (Bruker, 2003; Sheldrick, 2008), Fc^*^=kFc[1+0.001xFc^2^λ^3^/sin(2θ)]^-1/4^
0 constraints	Extinction coefficient: 0.103 (12)
Primary atom site location: structure-invariant direct methods	

### Fractional atomic coordinates and isotropic or equivalent isotropic displacement parameters (Å^2^)

**Table:**

	*x*	*y*	*z*	*U*_iso_*/*U*_eq_	
C1	0.3179 (2)	0.9158 (2)	0.76860 (15)	0.0474 (4)	
C2	0.3276 (2)	0.8319 (2)	0.64495 (16)	0.0560 (4)	
H2	0.3926	0.8952	0.5826	0.067*	
C3	0.2364 (3)	0.6502 (2)	0.61896 (18)	0.0641 (5)	
H3	0.2402	0.5898	0.5371	0.077*	
C4	0.1394 (3)	0.5554 (2)	0.71141 (19)	0.0658 (5)	
H4	0.0804	0.4330	0.6907	0.079*	
C5	0.1286 (3)	0.6392 (2)	0.83389 (17)	0.0572 (4)	
H5	0.0629	0.5747	0.8955	0.069*	
C6	0.2186 (2)	0.8227 (2)	0.86285 (15)	0.0477 (4)	
C7	0.2359 (2)	0.9517 (2)	0.97738 (15)	0.0477 (4)	
C8	0.3421 (2)	1.1130 (2)	0.95024 (15)	0.0491 (4)	
H8	0.3747	1.2189	1.0077	0.059*	
C9	0.1539 (2)	0.9133 (2)	1.10037 (16)	0.0526 (4)	
C10	0.5038 (3)	1.2371 (2)	0.76197 (16)	0.0563 (4)	
C11	0.5648 (3)	1.4194 (3)	0.8383 (2)	0.0756 (6)	
H11A	0.6392	1.4336	0.9248	0.113*	
H11B	0.4491	1.4377	0.8497	0.113*	
H11C	0.6452	1.5037	0.7894	0.113*	
C12	0.1351 (3)	1.0333 (3)	1.31867 (17)	0.0613 (5)	
H12A	0.1950	0.9683	1.3634	0.074*	
H12B	−0.0076	0.9667	1.3045	0.074*	
C13	0.1950 (3)	1.2107 (3)	1.40273 (18)	0.0724 (5)	
H13A	0.1508	1.1970	1.4875	0.109*	
H13B	0.1361	1.2743	1.3572	0.109*	
H13C	0.3366	1.2747	1.4175	0.109*	
N1	0.39555 (19)	1.09788 (17)	0.82406 (12)	0.0489 (4)	
O1	0.0559 (3)	0.76795 (18)	1.11828 (14)	0.0845 (5)	
O2	0.20161 (17)	1.05915 (15)	1.19112 (11)	0.0559 (3)	
O3	0.5434 (2)	1.20764 (19)	0.65295 (13)	0.0814 (5)	

### Atomic displacement parameters (Å^2^)

**Table:**

	*U*^11^	*U*^22^	*U*^33^	*U*^12^	*U*^13^	*U*^23^
C1	0.0485 (8)	0.0506 (9)	0.0420 (8)	0.0226 (7)	0.0046 (6)	0.0028 (6)
C2	0.0594 (10)	0.0612 (10)	0.0452 (9)	0.0262 (8)	0.0100 (7)	0.0006 (7)
C3	0.0707 (11)	0.0617 (11)	0.0531 (10)	0.0282 (9)	0.0083 (8)	−0.0092 (8)
C4	0.0744 (11)	0.0493 (10)	0.0660 (11)	0.0245 (9)	0.0072 (9)	−0.0049 (8)
C5	0.0631 (10)	0.0494 (9)	0.0552 (10)	0.0216 (8)	0.0084 (8)	0.0070 (7)
C6	0.0492 (8)	0.0497 (8)	0.0437 (8)	0.0231 (7)	0.0043 (6)	0.0036 (7)
C7	0.0521 (8)	0.0502 (9)	0.0419 (8)	0.0239 (7)	0.0077 (6)	0.0062 (7)
C8	0.0569 (9)	0.0503 (9)	0.0405 (8)	0.0245 (7)	0.0102 (6)	0.0032 (6)
C9	0.0617 (9)	0.0541 (10)	0.0467 (9)	0.0283 (8)	0.0122 (7)	0.0108 (7)
C10	0.0656 (10)	0.0570 (10)	0.0478 (9)	0.0254 (8)	0.0155 (7)	0.0131 (7)
C11	0.0980 (15)	0.0525 (11)	0.0726 (12)	0.0247 (10)	0.0284 (11)	0.0146 (9)
C12	0.0718 (11)	0.0789 (12)	0.0442 (9)	0.0395 (10)	0.0208 (8)	0.0160 (8)
C13	0.0812 (13)	0.0925 (14)	0.0491 (10)	0.0451 (11)	0.0156 (9)	0.0023 (9)
N1	0.0562 (8)	0.0484 (7)	0.0406 (7)	0.0215 (6)	0.0105 (5)	0.0044 (5)
O1	0.1286 (12)	0.0546 (8)	0.0692 (9)	0.0304 (8)	0.0424 (8)	0.0197 (6)
O2	0.0660 (7)	0.0586 (7)	0.0430 (6)	0.0256 (6)	0.0172 (5)	0.0069 (5)
O3	0.1158 (11)	0.0731 (9)	0.0556 (8)	0.0346 (8)	0.0377 (7)	0.0162 (7)

### Geometric parameters (Å, °)

**Table:**

C1—C2	1.389 (2)	C9—O1	1.197 (2)
C1—C6	1.399 (2)	C9—O2	1.3367 (19)
C1—N1	1.4186 (19)	C10—O3	1.201 (2)
C2—C3	1.379 (2)	C10—N1	1.400 (2)
C2—H2	0.9300	C10—C11	1.497 (3)
C3—C4	1.384 (3)	C11—H11A	0.9600
C3—H3	0.9300	C11—H11B	0.9600
C4—C5	1.381 (2)	C11—H11C	0.9600
C4—H4	0.9300	C12—O2	1.449 (2)
C5—C6	1.393 (2)	C12—C13	1.494 (3)
C5—H5	0.9300	C12—H12A	0.9700
C6—C7	1.449 (2)	C12—H12B	0.9700
C7—C8	1.352 (2)	C13—H13A	0.9600
C7—C9	1.467 (2)	C13—H13B	0.9600
C8—N1	1.391 (2)	C13—H13C	0.9600
C8—H8	0.9300		
			
C2—C1—C6	122.32 (15)	O2—C9—C7	112.43 (14)
C2—C1—N1	130.20 (15)	O3—C10—N1	120.26 (16)
C6—C1—N1	107.48 (13)	O3—C10—C11	123.11 (16)
C3—C2—C1	116.89 (17)	N1—C10—C11	116.63 (15)
C3—C2—H2	121.6	C10—C11—H11A	109.5
C1—C2—H2	121.6	C10—C11—H11B	109.5
C2—C3—C4	121.75 (17)	H11A—C11—H11B	109.5
C2—C3—H3	119.1	C10—C11—H11C	109.5
C4—C3—H3	119.1	H11A—C11—H11C	109.5
C5—C4—C3	121.27 (17)	H11B—C11—H11C	109.5
C5—C4—H4	119.4	O2—C12—C13	107.88 (15)
C3—C4—H4	119.4	O2—C12—H12A	110.1
C4—C5—C6	118.33 (17)	C13—C12—H12A	110.1
C4—C5—H5	120.8	O2—C12—H12B	110.1
C6—C5—H5	120.8	C13—C12—H12B	110.1
C5—C6—C1	119.44 (14)	H12A—C12—H12B	108.4
C5—C6—C7	133.47 (15)	C12—C13—H13A	109.5
C1—C6—C7	107.10 (14)	C12—C13—H13B	109.5
C8—C7—C6	107.50 (14)	H13A—C13—H13B	109.5
C8—C7—C9	126.52 (15)	C12—C13—H13C	109.5
C6—C7—C9	125.98 (15)	H13A—C13—H13C	109.5
C7—C8—N1	110.33 (14)	H13B—C13—H13C	109.5
C7—C8—H8	124.8	C8—N1—C10	126.27 (14)
N1—C8—H8	124.8	C8—N1—C1	107.60 (13)
O1—C9—O2	123.51 (15)	C10—N1—C1	126.12 (13)
O1—C9—C7	124.06 (16)	C9—O2—C12	116.25 (13)
			
C6—C1—C2—C3	−0.9 (2)	C8—C7—C9—O1	177.82 (17)
N1—C1—C2—C3	−179.95 (15)	C6—C7—C9—O1	−2.6 (3)
C1—C2—C3—C4	0.1 (3)	C8—C7—C9—O2	−2.5 (2)
C2—C3—C4—C5	0.5 (3)	C6—C7—C9—O2	177.09 (13)
C3—C4—C5—C6	−0.2 (3)	C7—C8—N1—C10	179.14 (15)
C4—C5—C6—C1	−0.6 (2)	C7—C8—N1—C1	0.04 (17)
C4—C5—C6—C7	179.54 (17)	O3—C10—N1—C8	179.02 (16)
C2—C1—C6—C5	1.2 (2)	C11—C10—N1—C8	−1.0 (3)
N1—C1—C6—C5	−179.57 (13)	O3—C10—N1—C1	−2.1 (3)
C2—C1—C6—C7	−178.95 (14)	C11—C10—N1—C1	177.96 (15)
N1—C1—C6—C7	0.31 (16)	C2—C1—N1—C8	178.95 (16)
C5—C6—C7—C8	179.58 (17)	C6—C1—N1—C8	−0.23 (16)
C1—C6—C7—C8	−0.29 (17)	C2—C1—N1—C10	−0.1 (3)
C5—C6—C7—C9	0.0 (3)	C6—C1—N1—C10	−179.32 (15)
C1—C6—C7—C9	−179.90 (14)	O1—C9—O2—C12	2.7 (2)
C6—C7—C8—N1	0.15 (18)	C7—C9—O2—C12	−177.03 (13)
C9—C7—C8—N1	179.76 (14)	C13—C12—O2—C9	−177.54 (14)

### Hydrogen-bond geometry (Å, °)

**Table:**

*D*—H···*A*	*D*—H	H···*A*	*D*···*A*	*D*—H···*A*
C2—H2···O3^i^	0.93	2.61	3.296 (2)	131
C5—H5···O1^ii^	0.93	2.64	3.273 (2)	125
C12—H12B···Cg1^iii^	0.96	2.95	3.618 (3)	127
C13—H13B···Cg2^iii^	0.96	2.78	3.587 (3)	142

Symmetry codes: (i) −*x*+1, −*y*+2, −*z*+1; (ii) −*x*, −*y*+1, −*z*+2; (iii) −*x*, −*y*+2, −*z*+2.
